# Normothermic Machine Perfusion of Explanted Human Metabolic Livers: A Proof of Concept for Studying Inborn Errors of Metabolism

**DOI:** 10.1002/jimd.70010

**Published:** 2025-03-03

**Authors:** Samira Safarikia, Riccardo Cirelli, Gionata Spagnoletti, Diego Martinelli, Giulia Bravetti, Paola Francalanci, Annamaria D'Alessandro, Giovina Di Felice, Marta Maistri, Elena Baldissone, Alberto M. Fratti, Raffaele Simeoli, Elisa Sacchetti, Sara Cairoli, Cristiano Rizzo, Rosanna Pariante, Michele Vacca, Andrea Cappoli, Christian Albano, Andrea Pietrobattista, Marco Spada, Carlo Dionisi Vici

**Affiliations:** ^1^ Research Unit of Clinical Hepatogastroenterology and Transplantation, Bambino Gesù Children's Hospital IRCCS Rome Italy; ^2^ Division of Hepatobiliopancreatic Surgery, Liver and Kidney Transplantation, Bambino Gesù Children's Hospital IRCCS Rome Italy; ^3^ Division of Metabolic Diseases and Hepatology, Bambino Gesù Children's Hospital IRCCS Rome Italy; ^4^ Cardiac Surgery Unit, Bambino Gesù Children's Hospital IRCCS Rome Italy; ^5^ Division of Pathology, Bambino Gesù Children's Hospital IRCCS Rome Italy; ^6^ Clinical Analysis Laboratory, Bambino Gesù Children's Hospital IRCCS Rome Italy; ^7^ Division of Anesthesiology and Intensive Care, Bambino Gesù Children's Hospital IRCCS Rome Italy; ^8^ Division of Transfusion Medicine, Bambino Gesù Children's Hospital IRCCS Rome Italy; ^9^ Division of Nephrology, Bambino Gesù Children's Hospital IRCCS Rome Italy; ^10^ B Cell Research Unit, Immunology Research Area, Bambino Gesù Children's Hospital IRCCS Rome Italy; ^11^ Unit of Hepatology and Transplant Clinic, Bambino Gesù Children's Hospital IRCCS Rome Italy

**Keywords:** machine perfusion, metabolic liver disease, pediatric liver transplantation

## Abstract

The human liver plays a central metabolic role; however, its physiology may become imbalanced in inborn errors of metabolism (IEM), a broad category of monogenic disorders. Liver transplantation has been increasingly used to improve patient metabolic control, especially in diseases related to amino acid metabolism, such as urea cycle disorders and organic acidurias, to provide enzyme replacement. Ex vivo liver normothermic machine perfusion (NMP) techniques have recently been developed to increase the number of transplantable grafts and improve transplantation outcomes. This study used seven NMP of explanted livers from patients with IEM undergoing transplantation as models to investigate disease‐related liver metabolism and function. The perfused livers demonstrated positive viability indicators and disease‐specific targeted metabolomics providing the proof‐of‐principle that our ex vivo model expresses the biochemical disease characteristics and responds to therapeutical intervention in a unique “physiological” milieu, offering an ideal tool to study novel treatments, in a setting closely mirroring human disease.

**Abbreviations**
3OHP: 3‐hydroxy propionate
ASA: argininosuccinic aciduria
C3: propionyl carnitine
CPS1: carbamoyl phosphate synthetase I
HA: hepatic artery
H&E: hematoxylin–eosin
IEM: inborn error of metabolism
MCA: methyl citrate
NMP: normothermic machine perfusion
NO: nitric oxide
OA: organic aciduria
OAT: ornithine aminotransferase
PA: propionic acidemia
PAS: periodic acid‐Schiff
PG: propionyl glycine
PV: portal vein
SCS: static cold storage
UCD: urea cycle disorders

## 1 Introduction

The liver plays a central metabolic role in the human body, as it is involved in several hundred physiological processes, including synthesis, detoxification, degradation, regulation, processing, and cellular trafficking machinery [1]. The tightly regulated liver physiology, which combines anabolic and catabolic processes, may become imbalanced due to an inborn error of metabolism (IEM), a broad category of monogenic disorders affecting metabolic and cellular pathways [2]. Cellular and animal models have helped understand the underlying pathological mechanisms of many IEMs, driving the discovery of new therapies. The generation of induced pluripotent stem cells and organoids has provided promising tools to study urea cycle disorders (UCD) and organic aciduria (OA), two IEM categories involved in amino acid metabolism [3, 4, 5, 6].

Although in vitro and animal models have played a crucial role in the study of IEMs, direct exploration of human metabolism is challenging because of the diverse expression of metabolic processes in tissues and subcellular organelles, often limiting our understanding of “metabolic compartmentalization” [7, 8, 9]. Recently, culturing precision‐cut liver slices has been successfully used to investigate ex vivo the impact of novel therapies in UCD [10].

In recent years, to increase the pool of transplantable organs and preserve and improve their viability, extracorporeal machine perfusion has emerged as a valuable tool to tackle these challenges, evolving from experimental technology to clinical standards [11, 12, 13, 14]. Various normothermic machine perfusion (NMP) techniques that allow ex vivo liver preservation even for prolonged periods have been developed [15, 16, 17, 18, 19].

However, the versatility of machine perfusion extends beyond transplantation, providing an ex vivo platform for disease study [20, 21, 22], for repair/reconditioning of damaged livers [23, 24, 25, 26, 27, 28, 29, 30] and to explore biomarkers linked to ex vivo liver function [31, 32, 33, 34].

As for IEMs, liver transplantation has been increasingly used, especially in diseases related to amino acid metabolism, such as UCD and OA, aiming to improve metabolic control, reduce disease burden, and prevent long‐term complications [35, 36], by replacing the underlying enzymatic defect. Remarkably, the affected IEM livers, explanted during transplantation, maintain their functional and structural integrity, allowing their use for domino transplantation [37].

This study aimed to address the use of NMP in livers explanted from patients undergoing transplantation for metabolic disorders, as a novel ex vivo model for IEM study.

## 2 Materials and Methods

This study was conducted at Bambino Gesù Children's Hospital of Rome, Italy. Livers were obtained from donors with UCDs and OA who underwent liver transplantation. Only livers not considered suitable for domino transplantation were used in the study, following an ethical protocol approved by the Hospital Ethical Committee (1522_OPBG_2018). Informed consent for the study was obtained at the time of obtaining consent for liver transplantation.

### 2.1 Liver Preparation and NMP

After total hepatectomy, the livers were cold‐perfused with Celsior solution (Celsior, IGL, France), weighed, and cannulated on the back table through the portal vein (PV), hepatic artery (HA), and bile duct. Following static cold storage (SCS), the livers were connected to a perfusion machine (Liver Assist, X‐Vivo, Mölndal, Sweden) for oxygenated NMP. Perfusion pressures were set to 80–100 mmHg for the hepatic artery (HA) and 10–15 mmHg for the portal vein (PV), with the aim of achieving a flow of 100 mL/min/100 g of liver parenchyma (25% through HA and 75% through PV).

The perfusion solution contained human AB0‐matched red blood cells, fresh frozen plasma, saline solution, albumin, essential nutrients, antibiotics, heparin, and electrolytes (Table S1). The hematocrit (HCT) was targeted at 25%–27%.

Donors estimated standard liver volume was calculated, based on the formula proposed by Herden et al. [38] and the ratio between the actual volume of the explanted and perfused liver and the estimated standard liver volume was calculated (Table 1).

**TABLE 1:** Characteristics of liver donors and duration of perfusions.

Sex	ASA‐1	ASA‐2	CPS‐1	PA‐1	PA‐2	PA‐3	PA‐4
	Female	Male	Male	Female	Male	Male	Female
Age at liver transplant (years)	7	19	2	1.1	10	16	1
Disease onset	Neonatal	Neonatal	Neonatal	Neonatal	Neonatal	6 months	Neonatal
Gene	*ASL*	*ASL*	*CPS1*	*PCCA*	*PCCA*	*PCCA*	*PCCB*
Allele 1	c. 436C>T	c. 1366C>G	c.1264‐2A>G	delex23	c.184_300del	c. 1268C>T	delex1_5
Allele 2	c. 436C>T	c. 1366C>G	c. 2975 T>C	delex23	c.184_300del	c. 1268C>T	delex1_5
Liver weight (g)	1395	1750	330	303	1130	1150	380
Estimated liver volume (mL)	642	1219	354	269	1092	1135	290
Actual‐to‐estimated liver volume ratio	2.17	1.44	0.93	1.13	1.03	1.01	1.31
SCS (min)	990	555	90	1080	420	70	750
NMP (h)	8.0	8.5	8.0	8.0	8.5	8.0	16

Abbreviations: ASA, argininosuccinic aciduria; CPS, carbamoyl phosphate synthetase I deficiency; NMP, normothermic machine perfusion; PA, propionic acidemia; SCS, static cold storage.

### 2.2 Perfusion Management and Parameters

Temperature, HA and PV pressures, flows and resistences were continuously monitored throughout the perfusion. A blood gas analyzer (GEM Premie 5000; Werfen, Bedford, MA, USA) was used to monitor lactate, pH, glucose, pO_2_ and pCO_2_. Glucose was targeted between 60 and 120 mg/dL and insulin was administered when needed. Oxygenation and acid–base balance were maintained at a pO_2_ of 100–200 mmHg and a pH of 7.3–7.45 through regulation of gas flow and bicarbonate administration. Arterial vasospasm was corrected via epoprostenol infusion (Caripul, Jessen Cilag S.P.A., Milano, Italy). Oxygen consumption was calculated using Fick's principle. Bile production was measured and analyzed for pH, HCO_3_^‐^, bilirubin, gamma‐glutamyl transferase, and LDH.

### 2.3 Laboratory Determinations

- Biochemical and hematological profile

Laboratory parameters (ALT, AST, gamma‐GT, ALP, ammonia, LDH, BUN, bilirubin, and electrolytes) were measured bihourly using a Roche cobas 8000 c 702 module (Basel, Switzerland). Red blood cell count, HCT and Hemoglobin (Hb) levels were measured using Siemens Advia 2120i (Munich, Germany), internal normalized ratio (INR), and factor V with STA R Max 2 (Diagnostica Stago S.A.S., Asnières sur Seine Cedex, France).

b Metabolic profile

Aminoacid analysis was performed using high‐performance liquid chromatography (1200 HPLC System, Agilent Technologies Inc., Santa Clara, CA, USA). 3‐hydroxypropionate (3OHP), propionyl glycine (PG), methylcitrate (MCA), and propionyl carnitine (C3) were assessed by tandem mass spectrometry using an API 4500 Qtrap Mass Spectrometer (Sciex, Framingham, MA, USA), as previously described [39]. Biomarker levels measured in the perfusate during NMP were compared with those observed in donors 12 months prior to transplantation.

c Biopsy analysis

Liver and bile duct biopsies were obtained before, during, and after perfusion, and analyzed after hematoxylin–eosin (H&E), periodic acid‐Schiff (PAS), cytokeratin 7, and CD31 dye staining.

### 2.4 Statistical Analysis

Graph‐Pad Prism 10 (Graph‐Pad Software Inc., San Diego, CA) was used for statistical analysis. Mean ± SD or median with interquartile range (IQR, 25th–75th percentiles) were used for normally distributed and not‐normally distributed measurements, respectively. Mann–Whitney and Kruskal–Wallis followed by Dunn's Multiple Comparison as post‐test were used as nonparametric tests to compare two or more groups, respectively. Two‐way ANOVA was used to evaluate changes among groups over time. A correlation matrix based on Spearman correlation r was also included to correlate nonparametric variables. The correlation coefficient ranges from −1 to +1, where +1 implies the existence of a linear equation establishing a relationship between two factors (X and Y) that increase simultaneously. The statistical significance was set at *p* < 0.05.

## 3 Results

### 3.1 Donors and Livers Characteristics

Seven livers from IEM patients were perfused, 6 for 8 h, and 1 for 16 h at a temperature between 36°C and 37.5°C. Donors demographics and livers characteristics are shown in Table 1. The mean liver weight was 919 ± 539 g, and the mean SCS duration before NMP was 565 ± 372 min. Data collection had some limitations. In the first perfused liver (PA‐1), the available data did not include lactate and metabolic analyses. Viability data of PA‐3 were not inserted in the analysis, as the HA could not be cannulated, and the perfusion was obtained only via the PV. Similarly, PA‐4 was evaluated only for the effect of glycine administration.

### 3.2 Vital Functions and Liver‐Related Profiles

**FIGURE 1:**
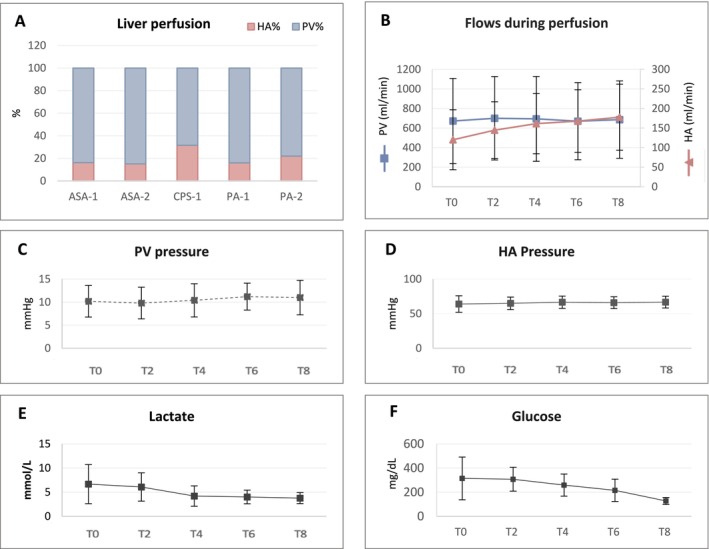
Hemodynamic and vitality parameters in the perfusate during normothermic machine perfusion. (A) Percent of liver perfusion provided by portal vein (PV) and hepatic artery (HA); (B) Perfusate flows through PV and HA (mean ± standard deviation); (C) PV pressure (mean ± standard deviation); (D) HA pressure (mean ± standard deviation); (E) Lactate levels (mean ± standard deviation); (F) Glucose levels (mean ± standard deviation).

Hepatocellular viability during perfusion was assessed in all livers. The PV flow was 692 ± 468 mL/min, corresponding to 69 ± 6 mL/min/100 g of liver parenchyma, whereas the HA flow was 156 ± 87 mL/min, corresponding to 18 ± 7 mL/min/100 g of liver parenchyma. Livers were perfused 80 ± 7% through the PV and 20 ± 7% through the HA (Figure 1A). PV flow remained stable, whereas HA flow significantly increased during perfusion (T0 120 ± 85 vs. T8 178 ± 94 mL/min, *p* < 0.01) (Figure 1B). In three cases, epoprostenol was added to maintain HA flow. The PV and HA pressures remained stable during perfusion (Figure 1C,D), allowing adequate oxygenation (Figure S1A–C) and facilitated CO_2_ extraction (T0 14 ± 10 vs. T8 31 ± 9 mmHg, *p* < 0.0001) (Figure S1D). The initial low pH was corrected by bicarbonate administration and maintained in the physiological range, while perfusate HCO_3_^−^ increased during perfusion (Figure S1E,F). Perfused livers demonstrated decreasing values of lactate (T0 6.7 ± 1.9 vs. T8 3.7 ± 1.1 mmol/L) (Figure 1E), while high glucose level at T0 (314 ± 177 mg/dL) was reduced at T8 (127 ± 129 mg/dL) (Figure 1F), requiring insulin administration in three livers.

**FIGURE 2:**
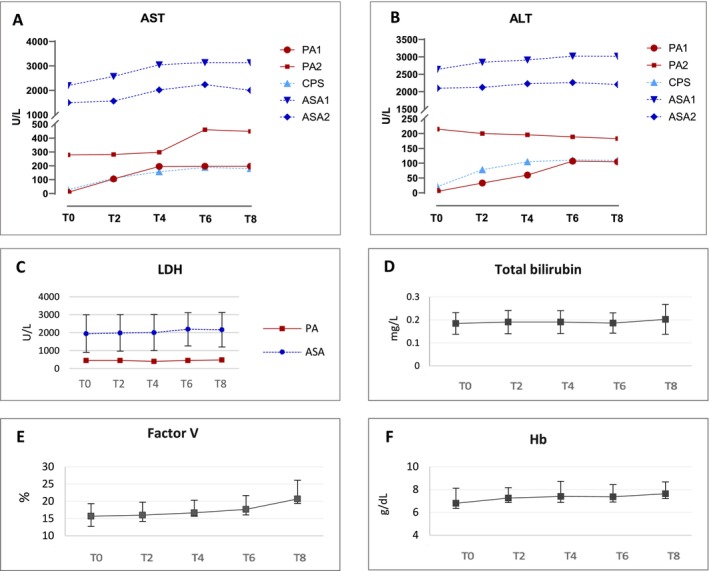
Functional and hematological parameters measured during normothermic machine perfusion. (A) Aspartate aminotransferase (AST); (B) Alanine aminotransferase (ALT); (C) Lactate dehydrogenase (LDH) in ASA livers (mean ± standard deviation) and in one PA liver; (D) Total bilirubin (mean ± standard deviation); (E) Factor V (mean ± standard deviation); (F) Hemoglobin (Hb) (mean ± standard deviation).

Transaminases and LDH were significantly elevated in ASA compared to non‐ASA livers (ALT 2537 ± 388 vs. 115 ± 69 U/L, *p* < 0.000; AST 2342 ± 613 vs. 209 ± 130 U/L, *p* < 0.0001; LDH 2060 ± 1051 vs. 446 ± 28 U/L, *p* < 0.001), maintaining stable values throughout NMP (Figure 2A–C). Gamma‐GT, total bilirubin, and ALP levels remained within the physiological ranges (Figure S2A,B). Factor V significantly increased (T0 15.7 ± 4.5 vs. T8 20.7 ± 6.6%, *p* < 0.05) with a concomitant decrease in INR (T0 3.97 ± 0.7, T8 2.7 ± 0.6; *p* < 0.01) (Figure 2E and Figure S2D), while hematological parameters remained within target ranges throughout perfusion (Figure S2C).

Bile production increased throughout perfusion (from 1.5 ± 0.3 to 4.7 ± 0.7 mL/h, *p* < 0.0001) (Figure S3A), with a cumulative volume of 9.4 ± 1.6 mL. The bile pH remained in an alkaline range, with increasing HCO_3_^−^ and bilirubin levels and decreasing gamma‐GT and LDH levels (Figure S3B–E). Bile quality was visually confirmed by its yellow‐greenish color (Figure S3F).

**FIGURE 3:**
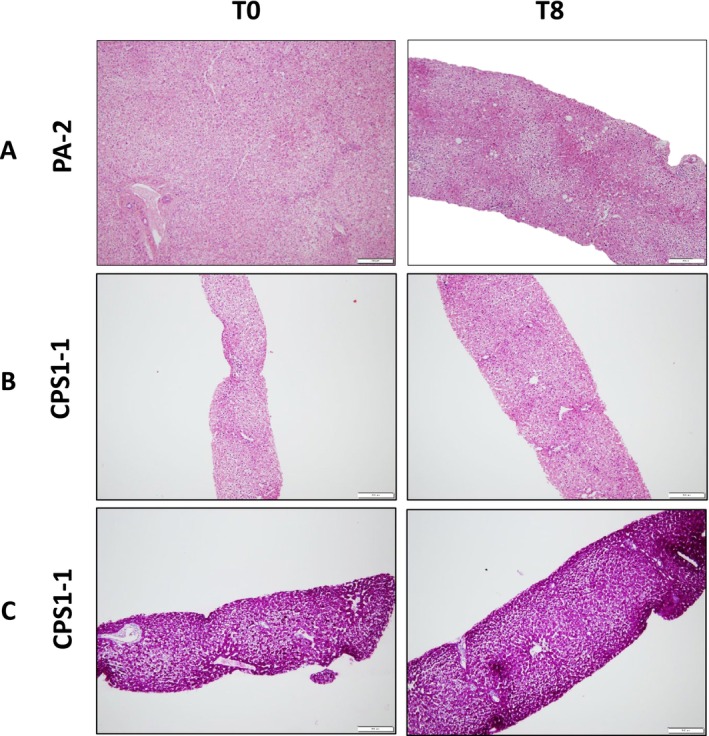
Livers histology before (T0) and after (T8) normothermic machine perfusion. (A) PA‐2: Preserved architecture with intact, viable hepatocytes; no apoptotic bodies (hematoxylin & eosin [H&E] staining; 10×); (B) CPS‐1: No pathological modifications at both T0 and T8 (H&E, 10×); (C) CPS‐1: No glycogen storage inside the hepatocytes (PAS, 10×).

The livers showed a preserved appearance in color, surface, and consistency (Figure S4A). Histological examination revealed that the architecture was preserved, and the hepatocytes were viable and intact. No intracellular glycogen inclusions or apoptosis were observed (Figure 3A–C). The sinusoids were patent (Figure S4C,D), without endothelial damage (Figure S4D). The bile ducts were normal in number, size, and position (Figure S5A–C). One liver showed incomplete septal cirrhosis in the context of native pathology (ASA) without further injury after perfusion (Figure S4B).

### 3.3 Disease‐Specific Metabolic Profiles

**FIGURE 4:**
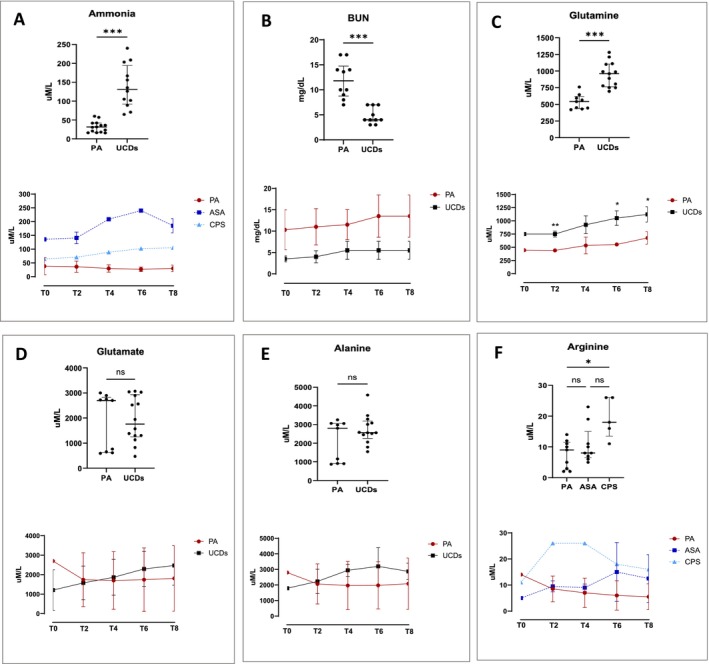
Metabolic parameters measured in the perfusate during normothermic machine perfusion. Top figures display median and interquartile range values of all measurements during perfusion (*p < 0.05; **p < 0.01; ***p < 0.001); bottom figures show the measurements at the different time points (mean ± standard deviation). (A) Ammonia levels in propionic acidemia (PA) and in urea cycle defects (UCDs) liver perfusions (top), variations during perfusion of PA, argininosuccinic aciduria (ASA) and carbamoyl phosphate synthetase 1 (CPS1) livers (bottom); (B) Urea nitrogen (BUN) levels in PA and in UCDs liver perfusions (top), variations during perfusion (bottom); (C) Glutamine levels in PA and in UCDs liver perfusions (top), variations during perfusion (bottom); (D) Glutamate levels in PA and in UCDs liver perfusions (top), variations during perfusion (bottom); (E) Alanine levels in PA and in UCDs liver perfusions (top), variations during perfusion (bottom); (F) Arginine levels in PA, ASA and CPS liver perfusions (bottom), variations during perfusion (bottom).

During NMP, ammonia levels were significantly different between the two disease categories, showing higher concentrations in UCD livers compared to PA livers (Figure 4A, top) with increasing values during perfusion (Figure 4A, bottom). Within the UCD category, ammonia levels were higher in ASA livers than in CPS1 livers. BUN levels (Figure 4B) were significantly higher in PA than those in UCD (top), with increasing values during perfusion (bottom).

The levels of glutamine (Figure 4C) were significantly elevated in UCD livers compared to PA livers (top), showing increasing values over perfusion (bottom). Overall, the perfusate concentrations of BUN and glutamine reflected those found in donors (Table 2). The glutamate values did not differ between the two disease categories (Figure 4D, top). However, like the glutamine profile, an increasing trend was recorded in UCD livers (Figure 4D, bottom). Notably, glutamate concentrations in the perfusate were about 30 times higher than those seen in the donors plasma (Table 2). Alanine levels were also elevated in the perfusate of both UCDs and PA livers (Figure 4E), with a 3‐6‐fold increase compared to donor plasma (Table 2).

**TABLE 2:** Plasma metabolic profiles of donors before liver transplantation. All values represent the mean ± standard deviation of measurements performed in the 12 months preceding transplantation, except for α‐fetoprotein and FGF21 which were determined only once before transplantation.

	ASA‐1	ASA‐2	CPS‐1	PA‐1	PA‐2	PA‐3	PA‐4
Ammonia (μM/L)	28 ± 15	88 ± 56	24 ± 17	21 ± 14	97 ± 35	26 ± 5	34 ± 10
BUN (mg/L)	2 ± 0.6	3 ± 0.7	9 ± 3	8 ± 3	10 ± 4	10 ± 6	7 ± 3
ALT (U/L)	104 ± 65	32 ± 9	50 ± 23	22 ± 5	35 ± 10	35 ± 15	33 ± 10
AST (U/L)	50 ± 15	21 ± 2	52 ± 26	37 ± 8	27 ± 7	31 ± 6	43 ± 10
Glutamine (μM/L)	735 ± 137	919 ± 92	755 ± 256	644 ± 197	784 ± 96	656 ± 42	360 ± 195
Glutamate (μM/L)	75 ± 17	76 ± 30	41 ± 15	72 ± 21	63 ± 22	39 ± 27	73 ± 30
Alanine (μM/L)	443 ± 52	535 ± 78	451 ± 117	363 ± 180	902 ± 247	582 ± 152	403 ± 202
Citrulline (μM/L)	227 ± 46	154 ± 32	41 ± 26	15 ± 7	21 ± 7	23 ± 5	18 ± 8
Arginine (μM/L)	91 ± 34^a^	65 ± 18^a^	87 ± 28^a^	56 ± 20	59 ± 21	49 ± 5	43 ± 27
Argininosuccinic acid (μM/L)	317 ± 129	479 ± 95	—	—	—	—	—
Glycine (μM/L)	373 ± 68	367 ± 106	210 ± 133	605 ± 261	1241 ± 199	842 ± 296	756 ± 338
Methylcitric acid (μM/L)	—	—	—	147 ± 68	182 ± 46	71 ± 35	55 ± 34
3OH‐propyonic‐acid (μM/L)	—	—	—	105 ± 5	186 ± 103	74 ± 2	7 ± 3
Propionyl carnitine (μM/L)	—	—	—	29 ± 20	28 ± 5	16 ± 6	34 ± 12
Propionyl glycine (μM/L)	—	—	—	13 ± 0.2	76 ± 23	34 ± 7	40 ± 36
α‐Fetoprotein (ng/mL)	3.1	4.4	5.9	17.0	11.5	3.6	33.6
FGF21 (pg/mL)	1523	2037	4178	494	2120	1019	2106

Abbreviations: ASA, argininosuccinic aciduria; CPS, carbamoyl phosphate synthetase I deficiency; PA, propionic acidemia; FGF, fibroblast growth factor.

^a^ While on oral arginine supplementation.

**FIGURE 5:**
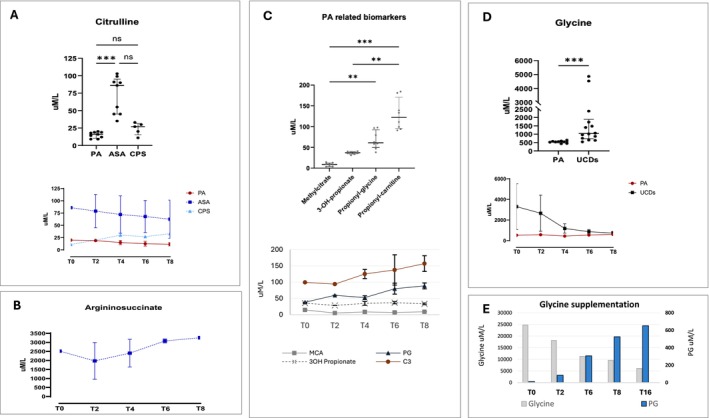
Metabolic parameters measured in the perfusate during normothermic machine perfusion. In A, C and D top figures display median and interquartile range values of all measurements during perfusion (*p < 0.05; **p < 0.01; ***p < 0.001); bottom figures show the measurements at the different time points (mean ± standard deviation). (A) Citrulline levels in propionic acidemia (PA), argininosuccinic aciduria (ASA) and carbamoyl phosphate synthetase 1 (CPS1) liver perfusions (top), variations during perfusion (bottom); (B) Argininosuccinic acid levels during perfusion of ASA livers (mean ± standard deviation); (C) Methylcitrate (MCA), 3‐hydroxy‐propionate (3OH propionate), propionyl‐glycine (PG) and propionyl‐carnitine (C3) levels in PA liver perfusions (top), variations during perfusion (bottom); (D) Glycine levels in PA and UCDs livers perfusions (top), variations during perfusion (bottom); (E) Glycine and PG levels during perfusion of PA‐4 liver after pre‐treatment (T0) of the perfusate solution with supraphysiological concentration of glycine.

Citrulline concentrations were higher in ASA livers than in CPS1 and PA livers (Figure 5A). Citrulline concentrations in ASA livers were significantly lower than those in the donors plasma (Table 2). Lower arginine levels were observed in the perfusate of PA and ASA livers compared to CPS1 (Figure 4F). The arginine concentrations in the PA perfusate were consistently lower than those in the donors plasma (Table 2). In perfused ASA livers, argininosuccinic acid levels showed an increasing trend over perfusion (Figure 5B), exceeding up to seven times those seen in donors plasma (Table 2).

Glycine concentrations (Figure 5D) were different between the two disease categories, with (unexpected) significantly higher values in UCDs than in PA (top). Interestingly, the highest glycine levels in UCD livers were recorded in the first 2 h of perfusion, followed by a decreasing trend over time, reaching levels like those of PA (bottom). In the PA perfusate, the mean glycine values (523 ± 67 μM/L) were lower than those observed in the donors plasma (Table 2). Regarding the levels of disease‐related biomarkers, C3 and PG were the major representative compounds in perfused PA livers, followed by 3OHP and MCA (Figure 5C, top). C3 (54%) and PG (28%) accounted for over 80% of the sum of the four biomarkers, whereas 3OHP (14%) and MCA (4%) accounted for less than 20%. C3 and PG showed increasing trend values, while 3OHP and MCA remained unchanged during perfusion (Figure 5C, bottom). The values and distribution of biomarkers in perfused livers strikingly diverged from those observed in donors plasma (Table 2), in which 3OHP (38%) and MCA (42%) accounted for 80% of the sum of the four biomarkers, with C3 (8%) and PG (12%) accounting for the remaining 20%.

To test the potential therapeutic use of glycine, the perfusion solution of one PA liver (PA‐4) was pre‐supplemented with a supraphysiological concentration of glycine. As shown in Figure 5E, during perfusion, glycine levels decreased, whereas PG levels significantly increased. The two compounds were highly correlated (*R* ^2^ = 0.9) with a net PG kinetic/production of 105 nmol/h/g liver.

### 3.4 Metabolic Correlations From the Spearman Correlation Test

As shown in Figure S6 (top), in UCD livers, ammonia, glutamine, glutamate, alanine, and BUN were positively correlated, while all these parameters were negatively correlated with glycine. In ASA livers (Figure S6 bottom), argininosuccinic acid was correlated with ammonia and glutamate and negatively with citrulline and glycine; arginine and citrulline showed opposite reciprocal correlations with disease‐related biomarkers. In PA livers (Figure S7), PG was positively correlated with glutamine, glycine, C3, and BUN, while negatively correlated with citrulline and arginine. Similarly, C3 was positively correlated with glutamine and BUN and negatively correlated with citrulline and arginine. MCA was positively correlated with glutamate, while 3OHP was not correlated with any of the markers. Ammonia was correlated with alanine, citrulline, and arginine, and negatively with BUN.

## 4 Discussion

In this study, we explored the possibility of creating a novel model for the ex vivo study of IEMs by applying the NMP technique to explanted livers from metabolic patients undergoing liver transplantation.

The perfused livers exhibited favorable functional, structural, and viability outcomes. Early hyperglycemia due to ischemia–reperfusion injury [40] decreased to normal levels with insulin supplementation, demonstrating an active liver hormonal response [41, 42]. Similarly, the initial high lactate levels, reflecting cold storage and lactate derived from packed red blood cells used in the perfusate [43], decreased during perfusion, providing valuable and earlier information on liver function than those provided by bile production and glucose metabolism/glucose measurement [20, 22, 23, 44]. Transaminase levels fulfilled the viability criteria [45, 46, 47], while the significantly higher levels in ASA livers confirmed the primary hepatic involvement in this disease [48]. Moreover, all the livers produced bile, indicating the recovery of secretory biliary function.

The few studies addressing the urea cycle and nitrogen related‐metabolism in “non‐metabolic” human livers showed, under different perfusion settings and analyzing different biological components (liver extracellular space by microdialysis or liver perfusate fluid) [34, 49, 50], that glutamate concentration was highly elevated compared to patients' plasma, while arginine, ornithine, and citrulline levels were significantly decreased in the backtable phase compared to the in situ state, with increasing concentrations after ex vivo perfusion. A recent untargeted metabolomics study confirmed the presence of higher ex situ glutamate levels, and RT‐qPCR analysis highlighted the upregulation of ornithine aminotransferase (OAT), ornithine transcarbamylase, and ornithine decarboxylases in ex situ liver samples [34], which may result in increased glutamate synthesis from α‐ketoglutarate, by shifting the OAT reaction toward the production of pyrroline‐5‐carboxylate. Our study, showing steadily elevated levels of glutamate in the perfusate of UCDs and PA livers, largely exceeding the concentration seen in the patient's plasma, confirms previous studies in non‐metabolic livers. Interestingly, in an experimental PA model, a major glutamate release was observed after perfusing rat livers with propionate and 3HP [51]. As seen in our model, a steady increase in BUN levels over perfusion time has been reported as a positive marker of liver function [34, 52].

Disease‐related biomarker analyses demonstrated, with some quantitative differences, that our ex vivo model closely mimicked what was observed in vivo in patients. Consistent with the decreased ureagenesis, ammonia and BUN levels in UCD differed significantly from PA livers. Accordingly, glutamine levels were higher in perfused UCDs livers than in PA livers, reflecting the increased glutamine synthesis to buffer the excess of ammonia [53]. Conversely, the lower glutamine level in perfused PA livers is consistent with the reduced availability of α‐ketoglutarate, causing decreased glutamine synthesis [54]. The lower levels of arginine and citrulline observed in our model confirm that they are primarily synthesized by the kidney and intestinal cells [55, 56]. Although the expected trend levels in the three disease models (i.e., ASA, CPS1, PA) were maintained, the absolute values of citrulline were lower than those measured in the patient plasma. Citrulline reduction could also be due to downregulation of the citrulline‐nitric oxide (NO) cycle. Under normal conditions, NO synthase utilizes L‐arginine and O_2_ to produce equimolar amounts of NO and L‐citrulline. However, ischemia–reperfusion injury can cause l‐arginine depletion, with a negative impact on NO and citrulline production [57, 58] and on microvascular flow [59]. Notably, administration of high doses of vasodilator was necessary to maintain the target HA flow in ASA‐perfused livers compared to other diseases. This demonstrates that perfused ASA livers express the characteristic defect in NO synthesis, due to the impaired ASL assembly with ASS, NOS, CAT1, and HSP90 in a functional multiprotein complex [60].

The levels of arginosuccinic acid in perfused livers were higher than those observed in ASA patients, reflecting not only its production but also its accumulation due to the absence of renal clearance during NMP.

The great differences in the distribution of the four PA‐related biomarkers between the ex vivo setting compared to that observed in vivo in patients advances our understanding of the role of the liver in detoxification processes. In contrast to the patient's plasma, C3 and PG were the major compounds in the perfusate, highlighting the primary function of the liver in the clearance of propionyl‐CoA, by producing its carnitine and glycine esters [61] through the action of carnitine acyl transferase and glycine‐N acylase [54]. Consistent with these findings, Guo et al. reported a significant increase in C3 levels also in perfused “non‐metabolic” livers [34].

To test the “druggability” of our model, glycine supplementation to a perfused PA liver demonstrated its conversion to PG to buffer the excess of propionyl‐CoA, highlighting the need to further explore in PA the potential therapeutic use of glycine, which represents a standard of care in isovaleric aciduria [62], thus far attempted only in a single patient [63].

Our study has some limitations. First, given the rarity of UCDs and OA, the sample size was limited. Second, we analyzed disease‐related biomarkers in a closed setting for a limited time. However, to allow a more comprehensive metabolic view in a prolonged time setting, NMP should be supported by dialysis (which provides the clearance of biomarkers), making necessary to analyze, in addition to the perfusate, liver extracellular fluid through microdialysis, and to extend the investigations to tissue expression studies (by mRNA and/or proteomic analyses) and/or fluxomics analyses using labelled molecules. Moreover, the recent possibility of simultaneous perfusion of split livers [19] provides a control organ for testing the impact of therapeutic interventions.

**FIGURE 6:**
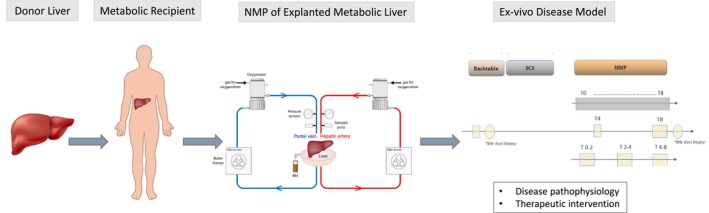
Schematic representation of ex vivo Normothermic Machine Perfusion of explanted metabolic livers. The flow chart illustrates sample collection of perfusate, bile fluid, hepatic and bile biopsies. NMP, normothermic machine perfusion; SCS, static cold storage.

In conclusion, this proof‐of‐concept model shows that the ex vivo NMP of metabolic livers (Figure 6) expresses the biochemical disease characteristics in a unique “physiological” milieu, providing novel insights into disease pathophysiology, on the distinct role of the liver in metabolic compartmentalization, and offering an ideal tool to study novel therapeutic interventions (e.g., mRNA, small molecules, cell therapies) in a setting closely mirroring the human disease.

## Author Contributions

C.D.V. and M.S. designed the experimental model and protocol and wrote the manuscript; S.F. contributed to the drafting of the protocol, coordinated the execution of the experiments and the analysis of the results, and wrote the manuscript; R.C., G.S., G.B., M.M., E.B., A.M.F., R.P., M.V., and A.C. participated in the execution of the experiments, data collection, and processing; and D.M., P.F., A.D.A., G.D.F., R.S., E.S., S.C., C.A., C.R., and A.P. participated in data collection and analysis. All authors revised and approved the final version of the paper.

## Consent

All procedures followed were in accordance with the ethical standards of the responsible committee on human experimentation (institutional and national) and with the Helsinki Declaration of 1975, as revised in 2000. Informed consent was obtained from all patients for being included in the study. Proof that informed consent was obtained will be available upon request.

## Conflicts of Interest

The authors declare no conflicts of interest.

## Supporting information


**Data S1.** Supporting Information.

## Data Availability

Data archiving is not mandated but data will be made available on reasonable request.
