# Supplementary material for: Normothermic Machine Perfusion of Explanted Human Metabolic Livers: A Proof of Concept for Studying Inborn Errors of Metabolism
Source: J Inherit Metab Dis. 2025 Mar 3;48(2):e70010. doi: 10.1002/jimd.70010 (PMC11874047; doi:10.1002/jimd.70010)
Supplement: Supplementary file 1 — Data S1. Supporting Information. [file JIMD-48-0-s001.pptx]

## Slide 1
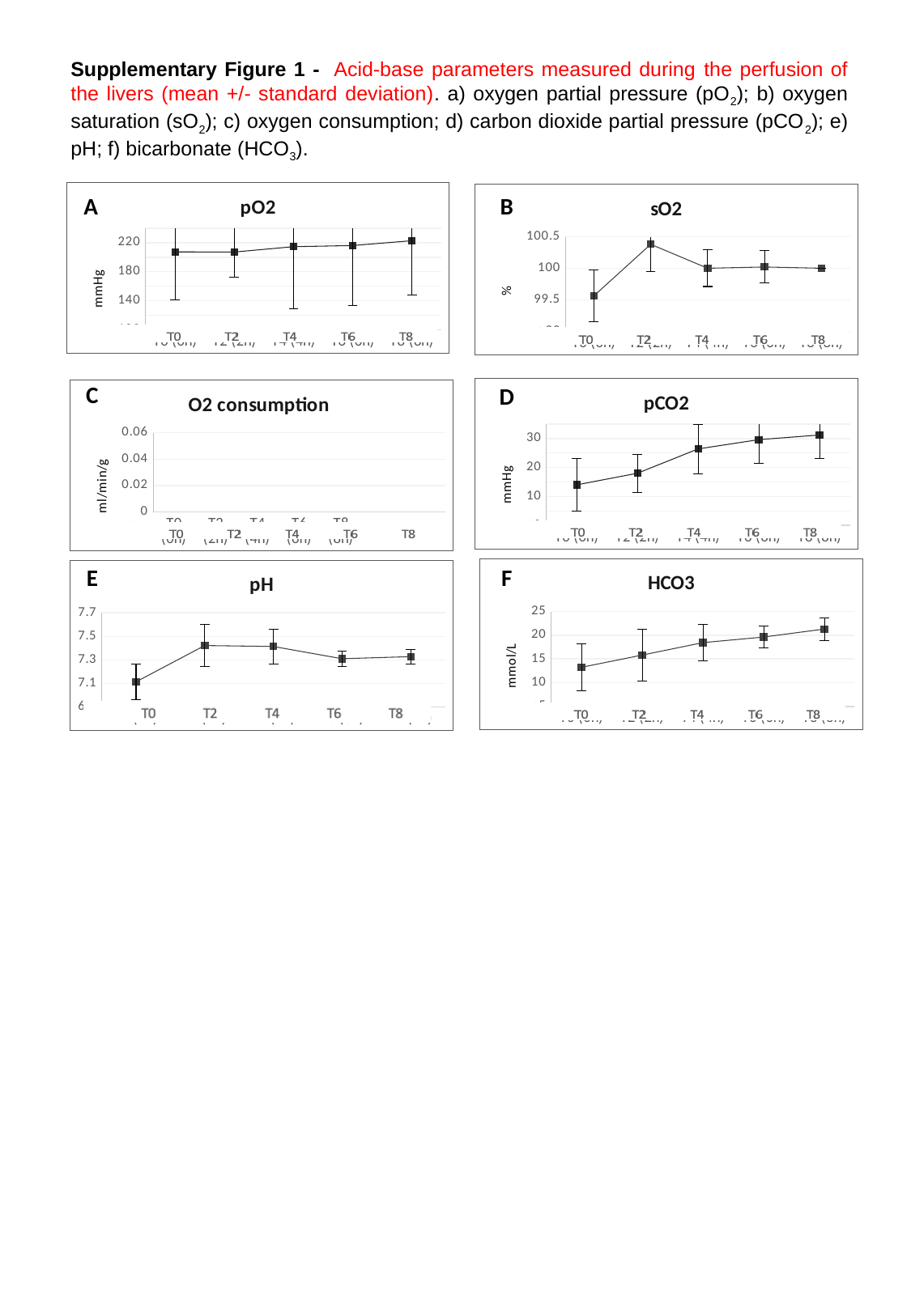

Supplementary Figure 1 - Acid-base parameters measured during the perfusion of the livers (mean +/- standard deviation). a) oxygen partial pressure (pO2); b) oxygen saturation (sO2); c) oxygen consumption; d) carbon dioxide partial pressure (pCO2); e) pH; f) bicarbonate (HCO3).
### Chart: pO2
| Category | 5 Livers Dual perfusion |
|---|---|
| T0 (0h) | 207.4 |
| T2 (2h) | 207.2 |
| T4 (4h) | 214.6 |
| T6 (6h) | 216.2 |
| T8 (8h) | 222.8 |
### Chart: sO2
| Category | 5 Livers Dual perfusion |
|---|---|
| T0 (0h) | 99.56 |
| T2 (2h) | 100.38 |
| T4 (4h) | 100.0 |
| T6 (6h) | 100.02000000000001 |
| T8 (8h) | 100.0 |A
B
C
D
### Chart: pCO2
| Category | 5 Livers Dual perfusion |
|---|---|
| T0 (0h) | 14.0 |
| T2 (2h) | 18.0 |
| T4 (4h) | 26.4 |
| T6 (6h) | 29.6 |
| T8 (8h) | 31.2 |
### Chart: O2 consumption
| Category | |
|---|---|
| T0 (0h) | 0.009793842325355802 |
| T2 (2h) | 0.024776737278580967 |
| T4 (4h) | 0.02465220958491016 |
| T6 (6h) | 0.03353080271036304 |
| T8 (8h) | 0.037717044844395656 |E
F
### Chart: HCO3
| Category | |
|---|---|
| T0 (0h) | 13.25 |
| T2 (2h) | 15.825 |
| T4 (4h) | 18.45 |
| T6 (6h) | 19.625 |
| T8 (8h) | 21.3 |
### Chart: pH
| Category | |
|---|---|
| T0 (0h) | 7.114 |
| T2 (2h) | 7.422 |
| T4 (4h) | 7.414 |
| T6 (6h) | 7.31 |
| T8 (8h) | 7.328 |

## Slide 2
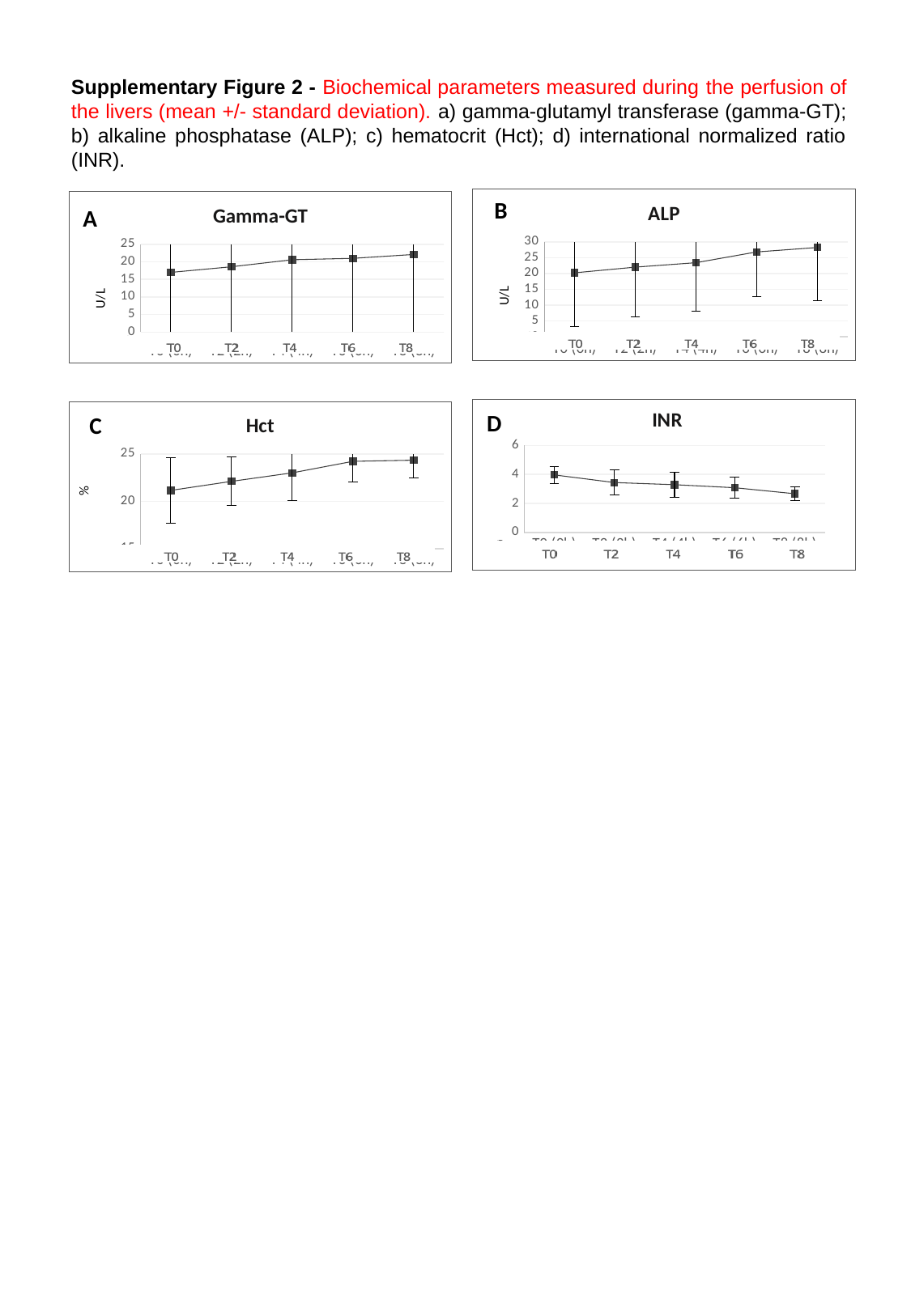

Supplementary Figure 2 - Biochemical parameters measured during the perfusion of the livers (mean +/- standard deviation). a) gamma-glutamyl transferase (gamma-GT); b) alkaline phosphatase (ALP); c) hematocrit (Hct); d) international normalized ratio (INR).
B
### Chart: ALP
| Category | 5 Livers Dual perfusion |
|---|---|
| T0 (0h) | 20.2 |
| T2 (2h) | 22.0 |
| T4 (4h) | 23.4 |
| T6 (6h) | 26.8 |
| T8 (8h) | 28.2 |
### Chart: Gamma-GT
| Category | 5 Livers Dual perfusion |
|---|---|
| T0 (0h) | 17.0 |
| T2 (2h) | 18.6 |
| T4 (4h) | 20.6 |
| T6 (6h) | 21.0 |
| T8 (8h) | 22.1 |
A
### Chart: INR
| Category | |
|---|---|
| T0 (0h) | 3.9666666666666663 |
| T2 (2h) | 3.44 |
| T4 (4h) | 3.2866666666666666 |
| T6 (6h) | 3.08 |
| T8 (8h) | 2.6666666666666665 |
### Chart: Hct
| Category | 5 Livers Dual perfusion |
|---|---|
| T0 (0h) | 21.160000000000004 |
| T2 (2h) | 22.119999999999997 |
| T4 (4h) | 23.02 |
| T6 (6h) | 24.240000000000002 |
| T8 (8h) | 24.36 |D
C

## Slide 3
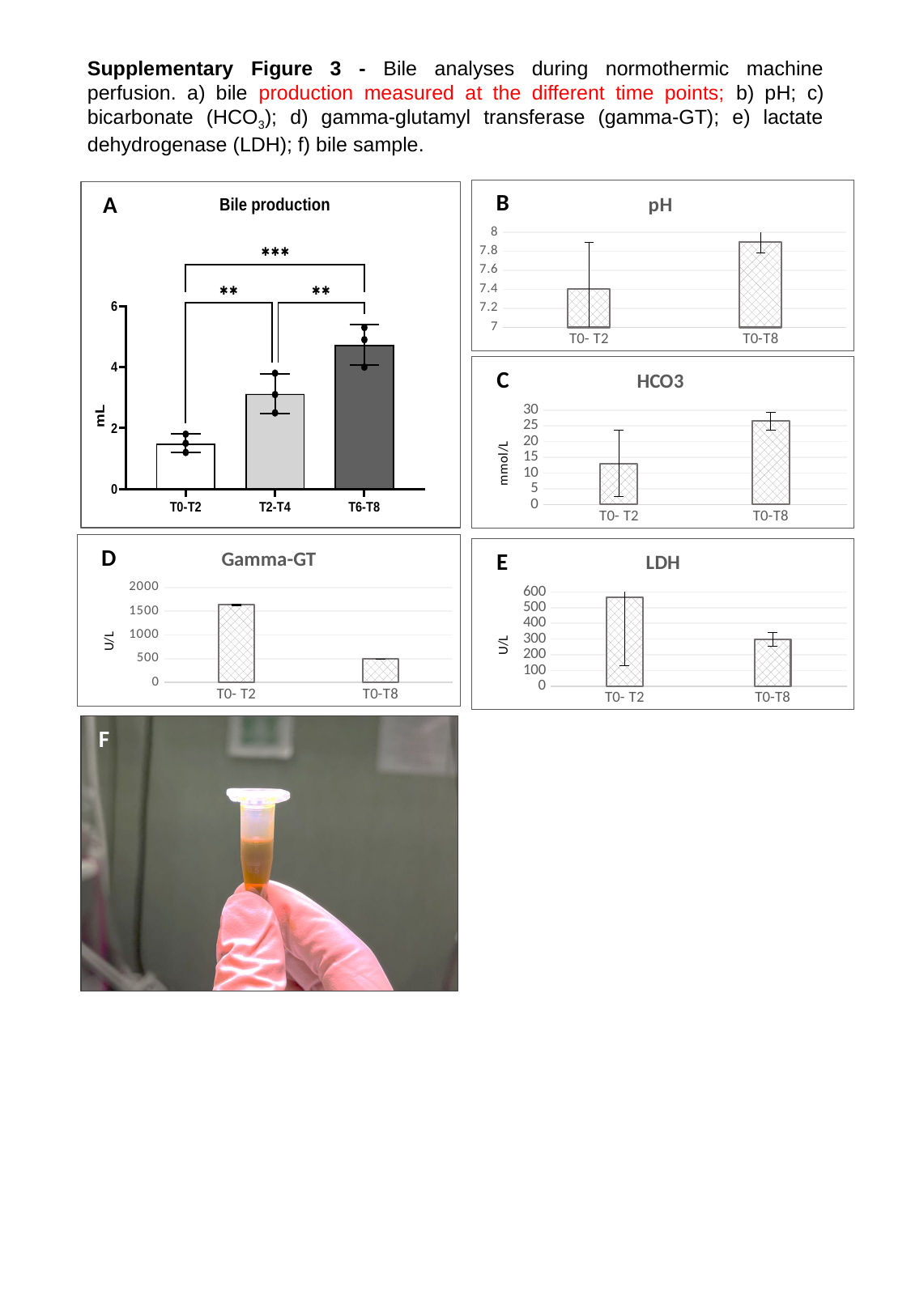

Supplementary Figure 3 - Bile analyses during normothermic machine perfusion. a) bile production measured at the different time points; b) pH; c) bicarbonate (HCO3); d) gamma-glutamyl transferase (gamma-GT); e) lactate dehydrogenase (LDH); f) bile sample.
a
b
### Chart: pH
| Category | |
|---|---|
| T0- T2 | 7.4 |
| T0-T8 | 7.9 |
### Chart: HCO3
| Category | |
|---|---|
| T0- T2 | 13.05 |
| T0-T8 | 26.5 |c
e
### Chart: LDH
| Category | |
|---|---|
| T0- T2 | 567.5 |
| T0-T8 | 299.0 |
d
### Chart: Gamma-GT
| Category | |
|---|---|
| T0- T2 | 1633.0 |
| T0-T8 | 490.5 |
F
B
A
C
D
E

## Slide 4
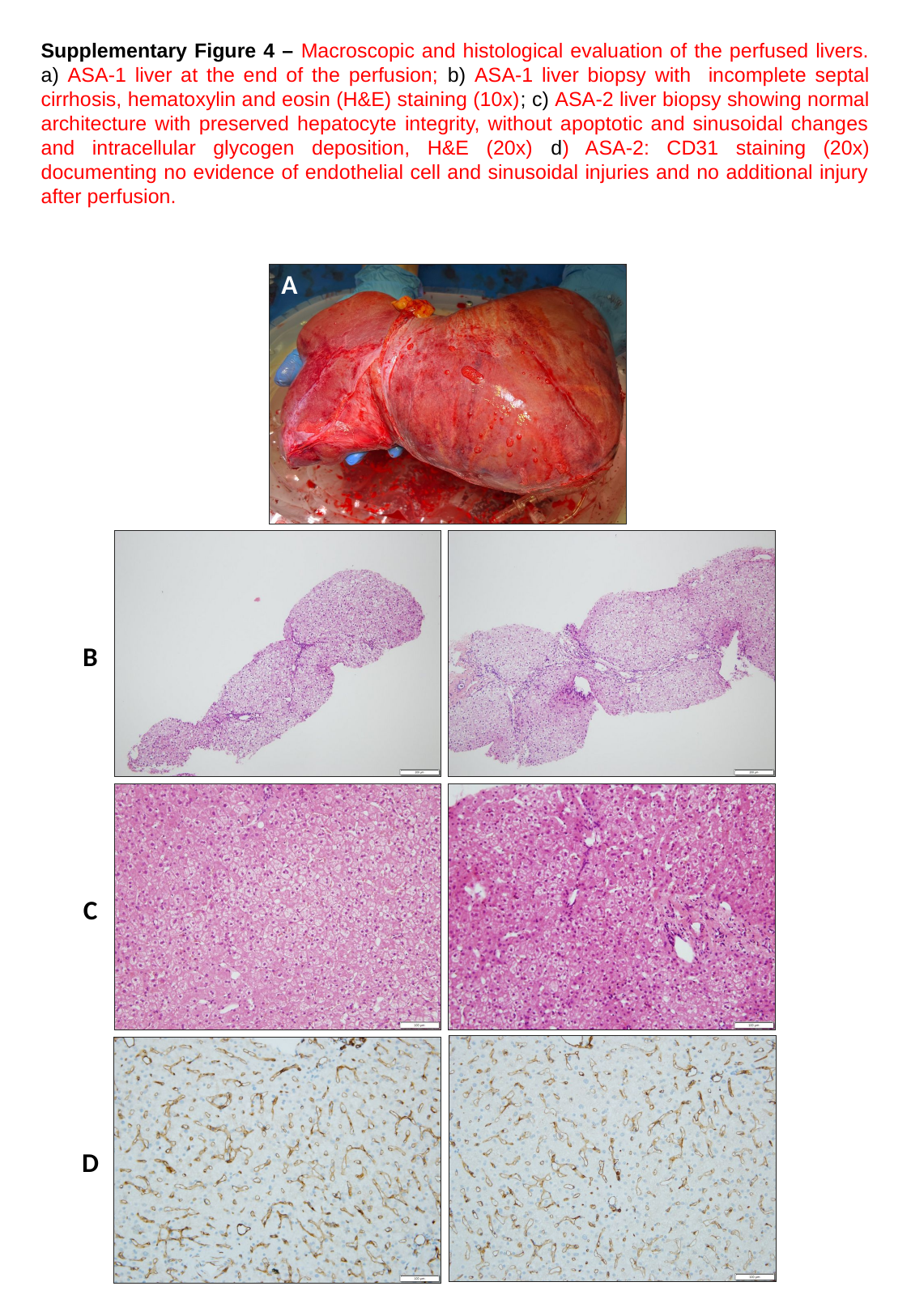

Supplementary Figure 4 – Macroscopic and histological evaluation of the perfused livers. a) ASA-1 liver at the end of the perfusion; b) ASA-1 liver biopsy with incomplete septal cirrhosis, hematoxylin and eosin (H&E) staining (10x); c) ASA-2 liver biopsy showing normal architecture with preserved hepatocyte integrity, without apoptotic and sinusoidal changes and intracellular glycogen deposition, H&E (20x) d) ASA-2: CD31 staining (20x) documenting no evidence of endothelial cell and sinusoidal injuries and no additional injury after perfusion.
A
B
C
D

## Slide 5
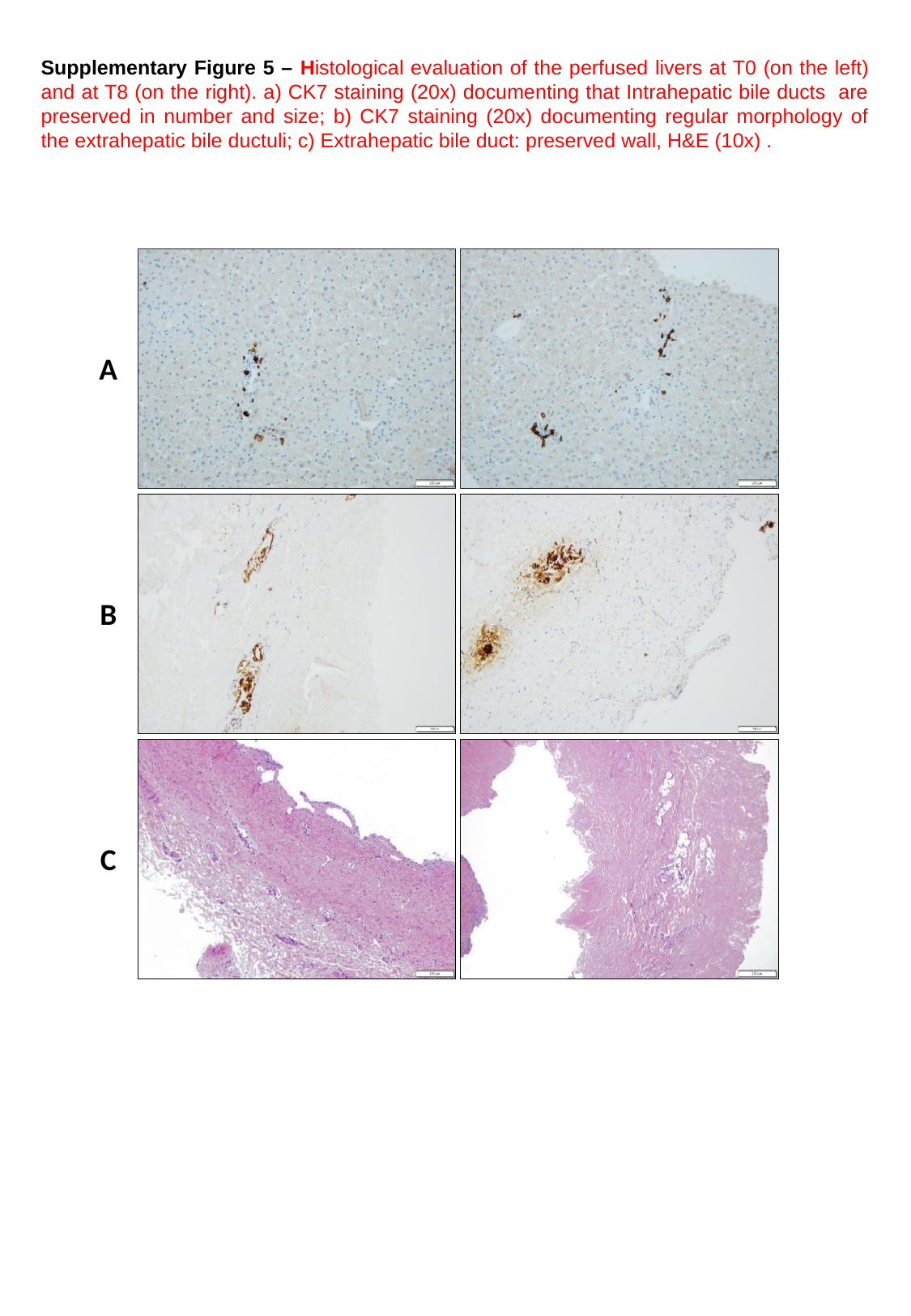

Supplementary Figure 5 – Histological evaluation of the perfused livers at T0 (on the left) and at T8 (on the right). a) CK7 staining (20x) documenting that Intrahepatic bile ducts are preserved in number and size; b) CK7 staining (20x) documenting regular morphology of the extrahepatic bile ductuli; c) Extrahepatic bile duct: preserved wall, H&E (10x) .
A
B
C

## Slide 6
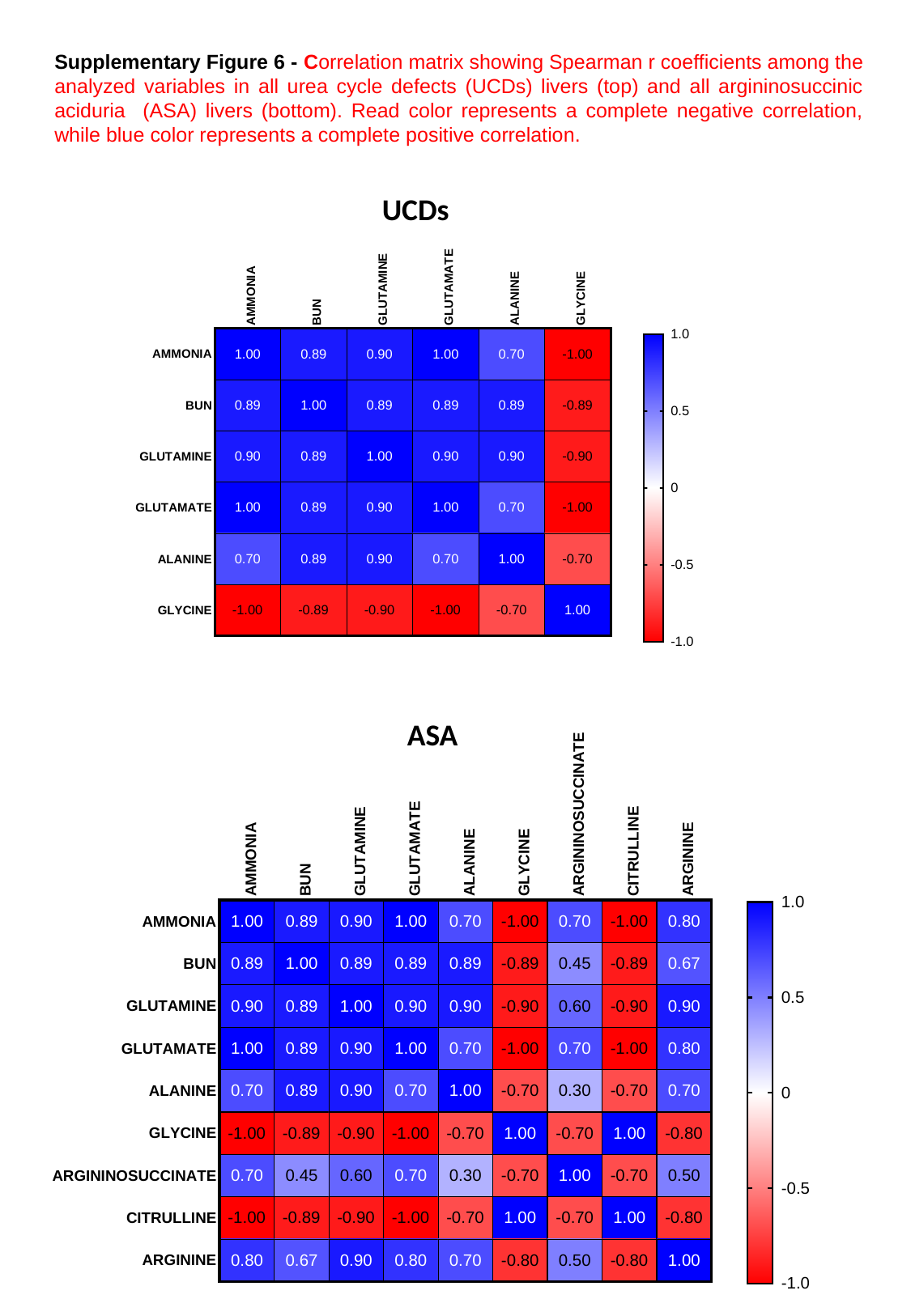

Supplementary Figure 6 - Correlation matrix showing Spearman r coefficients among the analyzed variables in all urea cycle defects (UCDs) livers (top) and all argininosuccinic aciduria (ASA) livers (bottom). Read color represents a complete negative correlation, while blue color represents a complete positive correlation.
UCDs
ASA

## Slide 7
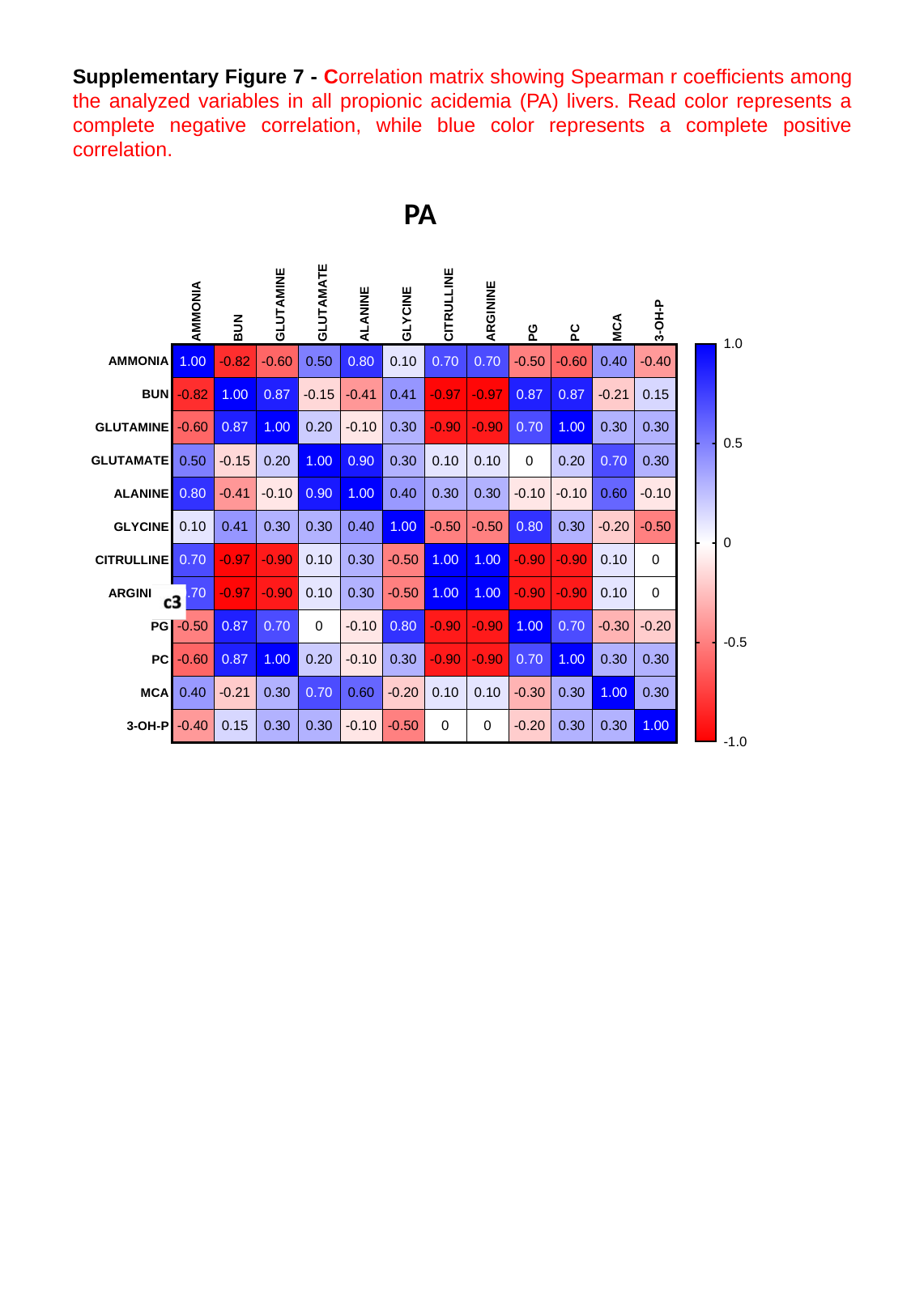

Supplementary Figure 7 - Correlation matrix showing Spearman r coefficients among the analyzed variables in all propionic acidemia (PA) livers. Read color represents a complete negative correlation, while blue color represents a complete positive correlation.
PA

## Slide 8
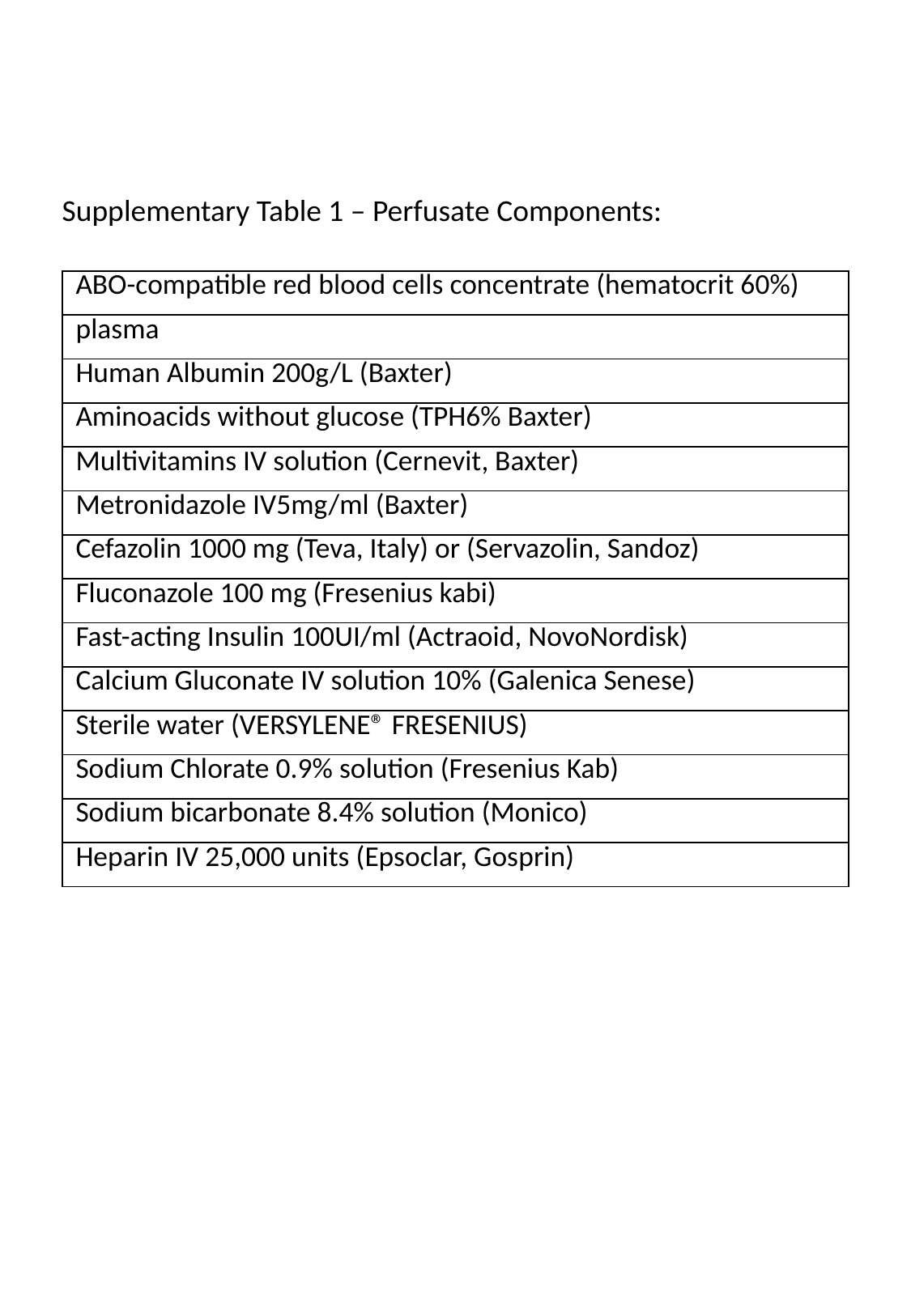

Supplementary Table 1 – Perfusate Components:
| ABO-compatible red blood cells concentrate (hematocrit 60%) |
| --- |
| plasma |
| Human Albumin 200g/L (Baxter) |
| Aminoacids without glucose (TPH6% Baxter) |
| Multivitamins IV solution (Cernevit, Baxter) |
| Metronidazole IV5mg/ml (Baxter) |
| Cefazolin 1000 mg (Teva, Italy) or (Servazolin, Sandoz) |
| Fluconazole 100 mg (Fresenius kabi) |
| Fast-acting Insulin 100UI/ml (Actraoid, NovoNordisk) |
| Calcium Gluconate IV solution 10% (Galenica Senese) |
| Sterile water (VERSYLENE® FRESENIUS) |
| Sodium Chlorate 0.9% solution (Fresenius Kab) |
| Sodium bicarbonate 8.4% solution (Monico) |
| Heparin IV 25,000 units (Epsoclar, Gosprin) |
